# miRinGO: Prediction of Biological Processes Indirectly Targeted by Human microRNAs

**DOI:** 10.3390/ncrna9010011

**Published:** 2023-01-22

**Authors:** Mohammed Sayed, Juw Won Park

**Affiliations:** 1 Division of Biomedical Informatics, Cincinnati Children’s Hospital Medical Center, Cincinnati, OH 45229, USA; 2 Department of Computer Science and Engineering, University of Louisville, Louisville, KY 40292, USA; 3 KBRIN Bioinformatics Core, University of Louisville, Louisville, KY 40292, USA; 4 CIEHS Biostatistics and Informatics Facility Core, University of Louisville, Louisville, KY 40292, USA

**Keywords:** microRNAs, transcription factors, gene ontology enrichment analysis, R Shiny application

## Abstract

MicroRNAs (miRNAs) are small non-coding RNAs that are known for their role in the post-transcriptional regulation of target genes. Typically, their functions are predicted by first identifying their target genes and then finding biological processes enriched in these targets. Current tools for miRNA functional analysis use only genes with physical binding sites as their targets and exclude other genes that are indirectly targeted transcriptionally through transcription factors. Here, we introduce a method to predict gene ontology (GO) annotations indirectly targeted by microRNAs. The proposed method resulted in better performance in predicting known miRNA-GO term associations compared to the canonical approach. To facilitate miRNA GO enrichment analysis, we developed an R Shiny application, miRinGO, that is freely available online at GitHub.

## 1. Introduction

Recent studies have suggested that microRNAs (miRNAs) are involved in many diverse biological processes and pathways including normal development and diseases [1]. Animal miRNAs bind to 3′UTR of mRNAs mainly through short sequences (6–8 NTs) called seed regions and act as repressors of target gene expression [2]. Taking into account this short sequence binding, one miRNA can target hundreds or even thousands of genes and subsequently perturb many biological pathways [3]. Given the cost of experimental identification of the function of a miRNA, potential miRNA-targeted pathways are first predicted computationally and then validated experimentally.

In order to computationally predict miRNA-targeted pathways, typically potential target genes are compiled using one or more miRNA target prediction tools and a standard gene enrichment analysis [4] is used to find potential enriched pathways or gene ontology (GO) terms. Although the conventional pipeline is widely used, it has some limitations. Of these, existing tools consider only direct targets (post-transcriptionally regulated) of miRNAs but do not consider indirect targets (mainly transcriptionally regulated) that are not necessarily enriched in miRNA seed-binding sites [5].

Indirect target genes are mainly regulated transcriptionally through transcription factors (TFs) [6,7]. Transcription factors are proteins that have the ability to regulate the transcription of other genes by binding to regions upstream of the gene sequence called promotors. Several studies have highlighted the interaction of miRNAs and TFs to modulate gene expression. Cui et.al., [8] showed that miRNA targets are enriched in nuclear proteins (mostly TFs) compared to other groups of cellular signaling genes such as ligands, cell-surface receptors and intracellular central signaling proteins. Shalgi et.al., [9] provided insight into the architecture of the miRNA-TF regulatory network. They found that TFs are often “target hubs” and are co-regulated by many miRNAs. Furthermore, they discovered frequent network motifs where miRNA-TF pairs co-regulate common genes and sometimes regulate each other; forming different types of feedforward/feedforward loops. Gosline et.al, [7] showed that in case of miRNA loss, changes in gene expression is dominated by indirect transcriptional effects rather than direct post-transcriptional effects and that TFs mediate and amplify miRNAs effects. 

Indirectly regulated genes can be enriched in a specific biological pathway or phenotype. One notable example is the role of the miR-200 family and miR-205 in controlling the epithelial to mesenchymal transition (EMT) pathway through targeting *ZEB1* and *ZEB2* transcription factors [10]. Jin et.al., [11] showed that four miRNAs Let-7g, Let-7a, miR-200a, and miR-375 regulate the cell cycle in pancreatic endocrine cells indirectly through cell cycle-related TFs such as E2F2. Other studies have shown how miRNAs regulate cell differentiation by targeting TFs. Of these, Tay et al. [12] demonstrated the role of miR-134 in embryonic stem cell differentiation by targeting *Nanog* and *LRH1*. Another study showed that miR-143 and miR-145 can work together to regulate smooth muscle cell differentiation and proliferation by targeting *KLF4* and *ELK1* transcription factors [13]. 

**Figure 1:**
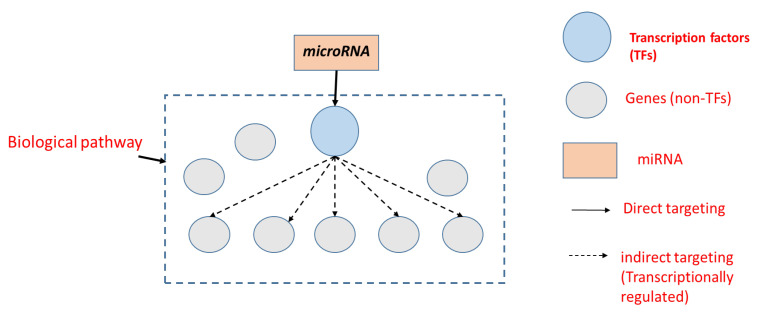
miRNAs can indirectly target biological pathways through transcriptions factors.

Figure 1 shows a scenario where biological pathways/processes can be missed by classical miRNA pathway analysis tools. In this scenario, the biological pathway consists of nine genes (one TF and eight non-TF). Using the classical method, the percentage of targeted genes is (1/9 = 11%), on the other hand, if we include TF targets (i.e., indirect targets), the percentage of targeted genes will be (6/9 = 67%) and this can make it more likely to be predicted as a target pathway.

Several tools and web servers have been developed to predict potential biological pathways targeted by miRNAs [14,15,16,17,18,19,20]. Of these, mirPath v3.0 [14], miTALOS [15], StarBase [16], and miRWalk v3.0 [17] are widely-used and their features are compared in Table 1. While they are all similar in terms of using direct targets only, they use different databases for both miRNA targets and gene ontology annotations [21,22,23,24,25]. miTALOS is the only tool that filters potential targets by incorporating tissue-specific genes. All tools except for StarBase accept multiple miRNAs as input. In this study, we introduce miRinGO (miRNA indirect target Gene Ontology) that uncovers potential biological pathways affected by indirect targets of human miRNAs especially ones related to cell differentiation and development. 

## 2. Methods

### 2.1. Overall Pipeline

**Figure 2:**
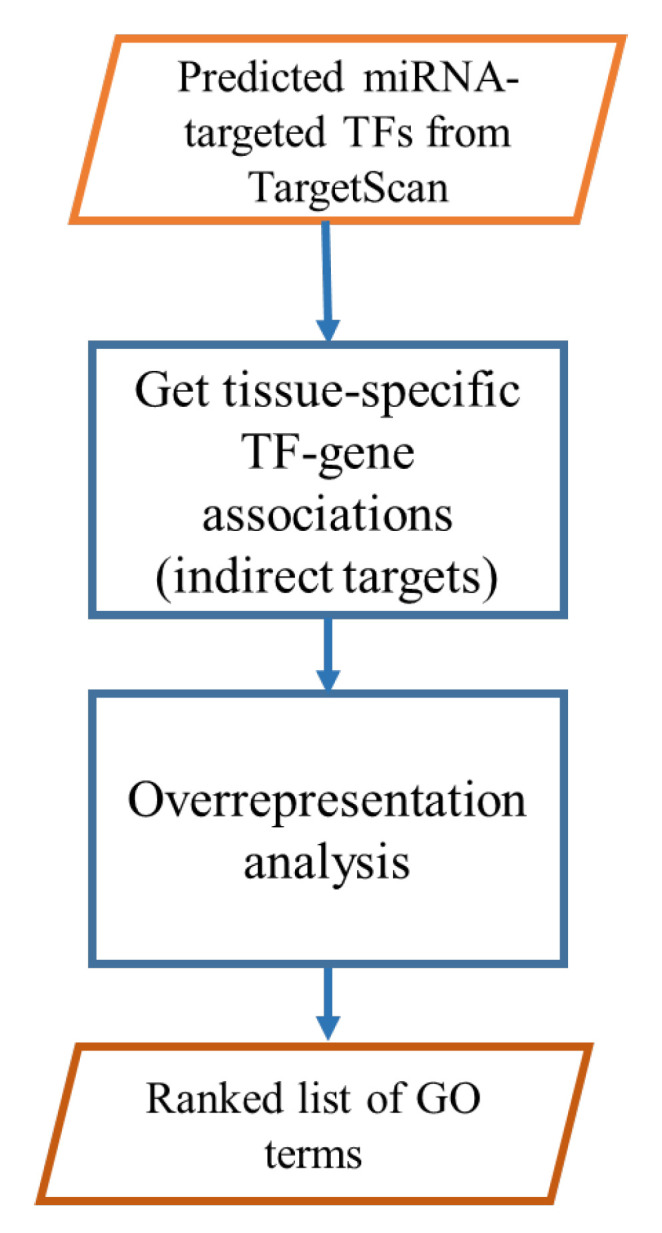
Pipeline of our miRNA GO enrichment analysis tool.

Our pipeline to predict indirectly targeted biological processes by miRNAs consists of three steps as depicted in Figure 2. First, for each miRNA, potential directly-targeted TFs were compiled from the TargetScan database v7.2 [26]. Second, computationally predicted tissue-specific TF-gene associations were collected from the resources website of (Sonawane et al., 2017) [27]. In the case of multiple input miRNAs, we use the intersection of indirect targets of each miRNA. Third, a hypergeometric test is conducted to find potential targeted biological processes. 

### 2.2. Input Data

Data used by our tool were compiled from publicly available databases. Putative miRNA targets were downloaded from TargetScan v7.2 [26]. We downloaded the file with all predictions regardless of the conservation of the miRNA family or miRNA binding sites and kept high-confidence human miRNA targets (*Cumulative weighted context++ score* <−0.1). 

Computationally predicted tissue-specific TF targets were downloaded from the resources website of (Sonawane et al., 2017) [27]. These TF targets were predicted using the PANDA (Passing Attributes between Networks for Data Assimilation) algorithm [28]. PANDA integrates three complementary sources of information, i.e., TF sequences motif data, protein-protein interactions of TFs and gene co-expression from Genotype-Tissue Expression (GTEx) RNA-Seq data [29]. It contains TF-gene associations from 38 different tissues/tissue locations. We aggregated TF-gene associations from different locations but belong to the same tissue. We had 29 broad tissues after aggregation.

Gene ontology annotations were downloaded using Ensembl Biomart [30] (version GRCh38). GO terms with less than five genes were removed.

### 2.3. Test Dataset

To validate our method, we used a ‘gold standard’ dataset of miRNAs and their experimentally validated functions (GO terms) [31] from ftp://ftp.ebi.ac.uk/pub/databases/GO/goa/HUMAN/goa_human_rna.gaf (accessed 15 January 2019). We filtered the dataset to include only high-confidence annotations (excluded annotations with “Inferred from Sequence or structural Similarity” (ISS), “Non-traceable Author Statement” (NAS), and “Traceable Author Statement” (TAS) evidence codes). We also removed annotations with no reference article. To keep only relevant annotations, we removed generic GO terms shared by most miRNAs (e.g., “miRNA mediated inhibition of translation”, “gene silencing by miRNA” and “gene silencing by RNA”). Cell/tissue ontology was downloaded from http://www.ontobee.org/listTerms/CL?format=tsv (accessed 15 January 2019). GO terms with less than five genes were removed. The filtered dataset consists of 335 pairs of miRNAs and their associated GO terms and is available in Supplementary Table S1.

## 3. Results

### 3.1. MicroRNA Indirect vs. Direct Targeting

**Figure 3:**
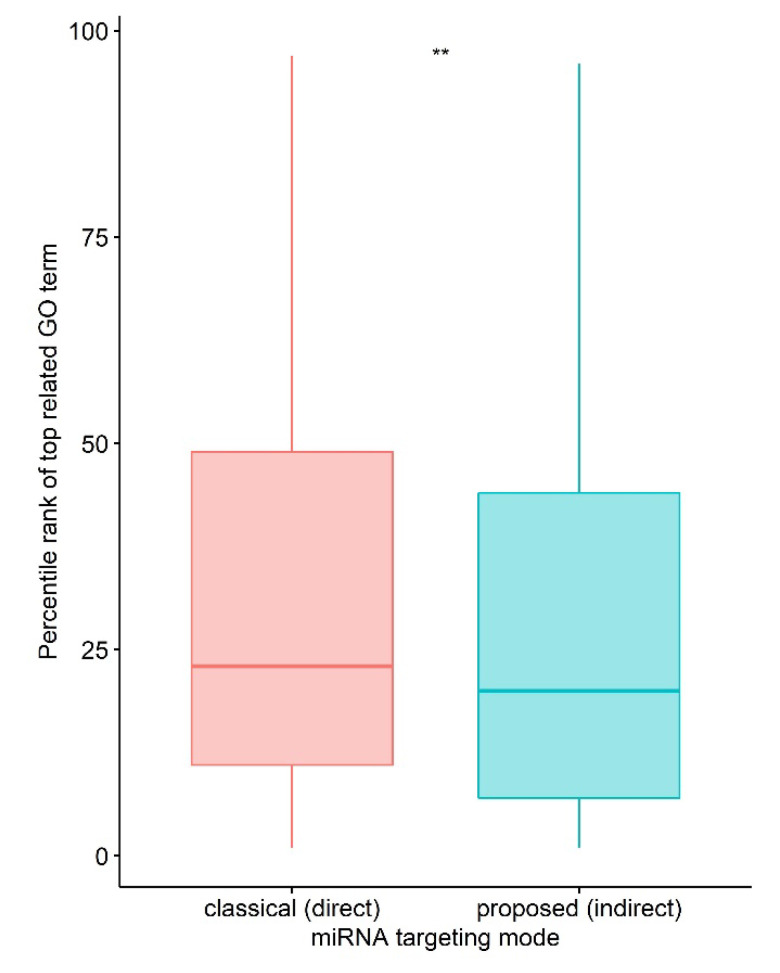
Comparison of indirect targeting with direct targeting (** represents *p*-value < 0.01).

To test the ability of our methodology to predict functions associated with a miRNA, we used a dataset with 335 known miRNA-GO term pairs. All TargetScan-predicted targets were included in this analysis. For each miRNA-GO term pair, resulting GO terms were ranked by the hypergeometric test *p*-value in ascending order, then rank values were converted to a percentile rank by dividing by the total number of GO terms. Finally, we picked the related GO term with the smallest *p*-value (smallest rank value). Known GO terms predicted by the indirect targeting method have a significantly lower (Wilcox signed-rank test, one-sided *p*-value = 0.002417) rank compared to canonical direct targeting as shown in Figure 3. 

### 3.2. Effect of Number of miRNA Targets

**Figure 4:**
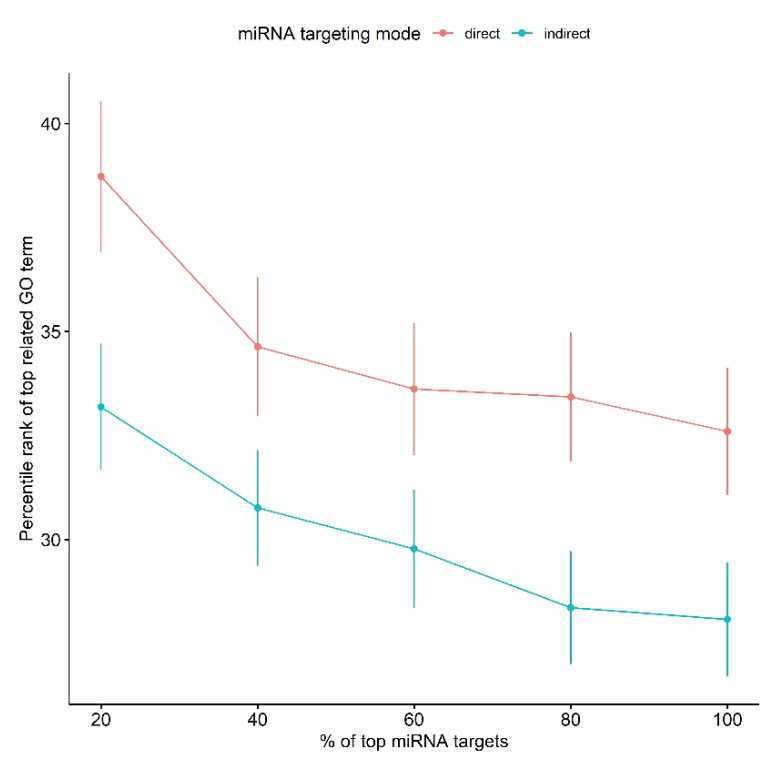
Effect of number of miRNA targets on miRNA GO enrichment analysis. Error bars represent one standard error.

Since miRNA GO enrichment analysis is affected by targets of the miRNA and TargetScan-predicted targets can have false positives, we investigated the effect of the number of predicted miRNA targets on predicting the known GO terms. We repeated the same analysis but instead of using all predicted targets, we used the top (20%, 40%, 60%, 80% and 100%) of potential targets (sorted by TargetScan context++ score [26]). Figure 4 shows that in all cases, the average percentile rank of GO terms predicted by the indirect targeting methodology is lower than those predicted by direct targeting. Although increasing the number of miRNA targets yielded a lower average rank (better performance), using all of the targets did not give significantly better results compared to using the top 80% of targets and 40% of targets in the case of indirect and direct targeting, respectively.

### 3.3. Indirect Targeting Reveals Role of miRNAs in Developmental Processes

To investigate biological processes that are more likely to be affected by indirect targeting of miRNAs, we calculated TF density per GO term as defined by Equation (1).

TF density = (Number of TFs in a GO term)/(Total number of genes in that term)(1)

Table 2 shows the top 5 GO BP terms with the highest TF density. All these GO terms are related to the “developmental process” and all genes involved are transcription factors.

**Figure 5:**
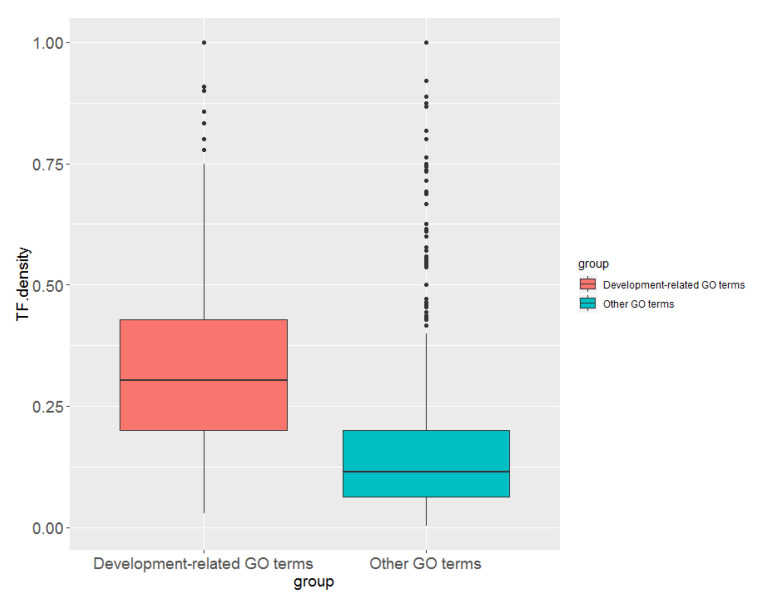
Comparison of TF density in development-related GO terms vs. all other terms.

To see if transcription factors are enriched in development-related GO terms compared to other terms, we divided the GO terms (that have at least one TF) into two groups; one with development-related terms and the second with other terms or processes. We selected development-related terms by searching for GO biological process terms with the following keywords (“development”, “cell fate”, “differentiation”, “stem cell”, “morphogenesis”, “cell specification”, “formation”). Figure 5 shows that development-related terms (*n* = 613) tend to have significantly (*p*-value < 2.2 × 10^−16^, Wilcoxon rank sum test) higher TF density compared to other terms (*n* = 1767).

### 3.4. Case Study: Role of miR-9 in Neurogenesis

To test the ability of our tool to capture relevant targeted development-related GO terms, we picked a miRNA with a known function to be able to compare our predicted GO terms with known ones. Of these miRNAs, miR-9 is a brain-enriched miRNA and has a prominent role in neurogenesis [32,33,34]. We ran our tool with the following inputs, we selected “brain” as the tissue type, “biological process” as the GO category, “indirect” as the targeting mode and “100” as the percentage of miRNA targets. Two out of the top five GO terms predicted are related to neurogenesis (“Nervous system development” and “brain development”). 

To compare our results with existing miRNA pathway analysis tools, we downloaded predicted GO biological process terms for “miR-9” from four different web servers (accessed January 15, 2019): mirPath v3.0 [14], StarBase (mirTarPathway module) v3.0 [16], miTALOS v2 [15] and miRWalk v3.0 [17]. We searched for the highest-ranking GO term related to neurogenesis as shown in Table 3. Our tool ranked neurogenesis-related GO terms higher than other tools.

### 3.5. Multiple miRNAs GO Analysis

In all miRNA GO analyses so far, we used one miRNA as an input. Several studies have shown that miRNAs can work together to regulate certain targets and biological processes [35,36]. Of these, Gregory et al. [10] showed that the miR-200 family and miR-205 together regulate epithelial-to-mesenchymal transition (EMT). The miR-200 family consists of miRNAs with two different seed sequences: miR-200a/miR-141 and miR-200b/miR-200c/miR-429. We ran our tool with the following inputs: “kidney” as the tissue type, “biological process” as the GO category, and “indirect” as the targeting mode and “100” as the percentage of miRNA targets. The rank of the *“epithelial to mesenchymal transition”* GO term (GO: 0001837) was lower when we used the intersection of indirect targets of these three miRNAs compared to ranks of GO terms predicted by each miRNA indirect target as shown in Table 4.

### 3.6. R Shiny Application

**Figure 6:**
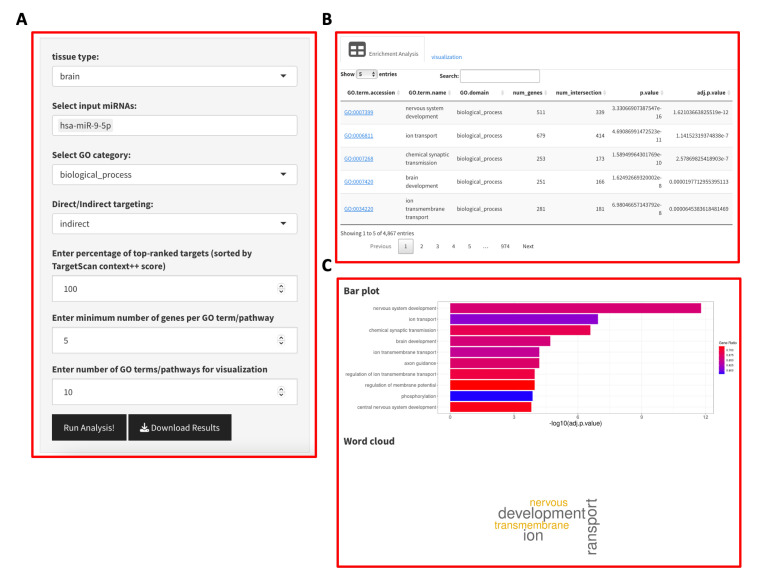
User interface of miRinGO R shiny application. (**A**) input parameters; (**B**) Table with miRNA GO enrichment analysis; (**C**) Bar plot and word cloud summarizing the top enriched GO terms.

For ease of use of our method, we developed a web application, miRinGO, using the R Shiny package [37]. The miRinGO user interface has two panels as shown in Figure 6, the left one for input data and parameters selection and the right one for displaying the miRNA GO enrichment results. The enrichment results are provided in a table format and top GO terms are visualized using bar plot and word cloud. Word cloud visualization summarizes the most frequent words in enriched GO terms where the size of the word is proportional to how many times we see this word. The tool provides users with the ability to choose different input data and parameters as detailed in Supplementary Table S2.

## 4. Discussion

We propose miRinGO, an easy-to-use tool that detects biological processes indirectly targeted by miRNAs transcriptionally through transcription factors. Using miRinGO, we can include potential target genes even if there is no physical interaction between miRNA and the regulated genes. In order to validate this method, we used a dataset of miRNAs and their known targeted GO terms [31]. Although this dataset is considered a significant step towards having a gold standard to validate different miRNA pathways or GO analysis tools, it is still limited to a fraction of human miRNAs and focused more on cardiovascular-related processes. Using this dataset, however, indirect targeting showed better performance in predicting known targeted processes compared to the direct targeting method, even if we use different fractions of input miRNA targets. It is also worth noting that although increasing the number of miRNA targets yielded better performance, using all of the targets did not give significantly better results compared to using the top 80% of targets and 40% of targets in the case of indirect and direct targeting, respectively. This could be due to the fact that miRNA target prediction tools suffer from having many false positives [37]. 

Since our method is mainly focused on miRNA-targeted TFs and development-related GO terms or pathways have more TFs than other terms, it is more suitable to use this tool to uncover the tissue-specific roles of miRNAs in development and cell differentiation. Using this method, we predicted biological pathways known to be targeted by miR-9, a miRNA with a known role in neurogenesis. Tan et al. [38] showed that miR-9 regulates neural stem cell differentiation and proliferation by targeting the *HES1* transcription factor. Using indirect targeting, three genes related to neuron differentiation (*FEZF2, SOX3* and *ZHX2*) that are predicted to be targeted by *HES1* (but are not direct targets of miR-9-5p) are now included in GO enrichment analysis as indirect targets of miR-9-5p.

One limitation of our method is that we use two sets of computationally predicted targets: one for miRNA direct targets and the other for tissue-specific TF targets. This might increase the effect of false positives in miRNA GO enrichment analysis. This limitation can be alleviated in the future by using (1) high-confidence miRNA targets (i.e., ones with smaller TargetScan *context++* score) (2) experimentally-validated miRNA targets from databases such as TarBase [39] and miRTarBase [40] (3) experimentally-validated TF targets supported by chromatin immunoprecipitation followed by deep-sequencing analysis (ChIP-seq) experiments. Although our method outperformed the current miRNA GO analysis method, it is not intended to replace the standard miRNA GO analysis method but on the other hand, to give a different perspective of miRNA roles in regulating biological processes and to uncover ones that are previously overlooked by current tools, especially ones related to development and cell differentiation. 

## Figures and Tables

**Table 1:** A comparison of the widely used tools of miRNAs pathway analysis.

Features/Tools	mirPath v3.0	StarBase	miTALOS	miRWalk v3.0
Predicted targets databases	TargetScan (v6)/microT-CDS (v5.0)	TargetScan/miRanda/PITA/RNA22/PicTar/…	TargetScan/miRanda	TargetScan (v7.1)/miRDB
Validated targets databases	TarBase v7.0	CLIP-Seq data	CLIP-Seq data	miRTarbase
Pathways/GO terms databases	KEGG/GO categories	KEGG/GO/Reactome/BioCarta	KEGG/WikiPathways/Reactome	KEGG/GO/Reactome
Inclusion of indirect targets?	No	No	No	No
Tissue specific?	No	No	Yes	No
Allows multiple miRNAs?	Yes	No	Yes	Yes

**Table 2:** Top 5 GO terms with the highest TF density.

GO Term ID	GO Term	Number of TFs	Number of Genes	Parent Process
GO:0001714	endodermal cell fate specification	5	5	developmental process
GO:0003211	cardiac ventricle formation	5	5	developmental process
GO:0003357	noradrenergic neuron differentiation	5	5	developmental process
GO:0021520	spinal cord motor neuron cell fate specification	7	7	developmental process
GO:0021902	commitment of neuronal cell to specific neuron type in forebrain	7	7	developmental process

**Table 3:** Comparison of highest-ranking GO terms related to neurogenesis from different miRNA GO enrichment tools.

Tool	Highest Ranking GO Term Related to Neurogenesis	Rank
miRinGO	Nervous system development	1
mirPath v3	Regulation of neuron maturation	11
miRWalk v3	Axonogenesis	13
StarBase v3	Neurogenesis	23
miTALOS v2	N/A	N/A

**Table 4:** Effect of using multiple miRNAs in capturing EMT-related GO terms.

miRNAs	Rank of Top GO Term Related to EMT
miR-200a/miR-141	132
miR-200b/miR-200c/miR-429	147
miR-205-5p	105
All three miRNAs	70

## Data Availability

miRinGO Source code and accompanying data is freely available from GitHub at https://github.com/Fadeel/miRinGO.

## Supplementary Materials

The following supporting information can be downloaded at: https://www.mdpi.com/article/10.3390/ncrna9010011/s1, Table S1: gold standard dataset of microRNAs and their experimentally validated gene ontology (GO) terms; Table S2: detailed description of the user interface of miRinGO R Shiny application.
